# Membranous nephropathy and thymoma in a patient with ankylosing spondylitis: A case report

**DOI:** 10.1097/MD.0000000000020111

**Published:** 2020-05-01

**Authors:** Lirong Lin, Lei Zhao, Bengang Huo, Luquan Zheng, Rongjie Yu, Weibing Li, Jurong Yang

**Affiliations:** Department of Urology, The Third Affiliated Hospital of Chongqing Medical University (Gener Hospital), Chongqing, China.

**Keywords:** ankylosing spondylitis, membranous nephropathy, thymoma

## Abstract

**Rationale:**

We report a rare case with ankylosing spondylitis (AS), thymoma, and membranous glomerulonephritis. The pathogenic mechanisms of these 3 diseases may be associated with each other. Here, we discuss the course of diagnosis and treatment.

**Patient concerns:**

A 64-year-old woman with bilateral pain of the sacroiliac joints for 10 years and anasarca for 10 days.

**Diagnoses:**

A diagnosis of AS by HLA-B27 and pelvic X-ray tests, thymoma based on computed tomography and pathological diagnosis, and membranous glomerulonephritis based on renal biopsy.

**Interventions:**

We administered methylprednisolone 500 mg/d for 3 consecutive days, followed by methylprednisolone 40 mg oral QD, for a month.

**Outcomes:**

The patient was followed up once a month. In the sixth month, the patient's serum creatinine had decreased to 0.96 mg/dL, urine microalbumin/creatinine decreased to 173.3 mg/g, and albumin had risen to 33.1 g/L. Pain and morning stiffness were relieved, and the Bath Ankylosing Spondylitis Disease Activity Index score dropped to 4.0.

**Lessons:**

Although the causal relationship between AS, thymoma, and membranous nephropathy in this patient still needs to be established, the pathogenesis between the 3 diseases may have some association. In clinical practice, patients with AS need to be screened for tumors and renal complications.

## 1 Introduction

Ankylosing spondylitis (AS) is a chronic progressive autoimmune disease that mainly affects the sacroiliac joints, spine, and peripheral joints.^[1]^ It can lead to spinal deformity and ankylosis in severe cases.^[2]^ AS patients can have extra-articular manifestations such as renal involvement and tumors.^[3]^ The most common types of tumors in AS patients include hematological tumors and prostate, bone, and colon cancers. The more commonly reported renal complications are renal amyloidosis,^[4]^ IgA nephropathy,^[5]^ and tubulointerstitial nephropathy.^[6]^ To our knowledge, this current study is the first to report a case of an AS patient with both renal complications, namely membranous nephropathy (MN), and a tumor, namely a thymoma.

## 2 Case report

The patient was a 64-year-old woman who had anasarca for 10 days and proteinuria (++++). The patient had been diagnosed with AS 10 years earlier. Her symptoms included bilateral pain of the sacroiliac joints. HLA-B27 assay results had been positive. Radiographic studies showed signs of sacroiliitis. The pain was relieved by intermittent treatment with non-steroidal anti-inflammatory drugs. One year before presenting at the hospital, a chest computed tomography examination was performed for a complaint of productive cough, and a mediastinal mass was revealed. The mass was resected and a pathological diagnosis of thymoma (type A) was made.

**Figure 1:**
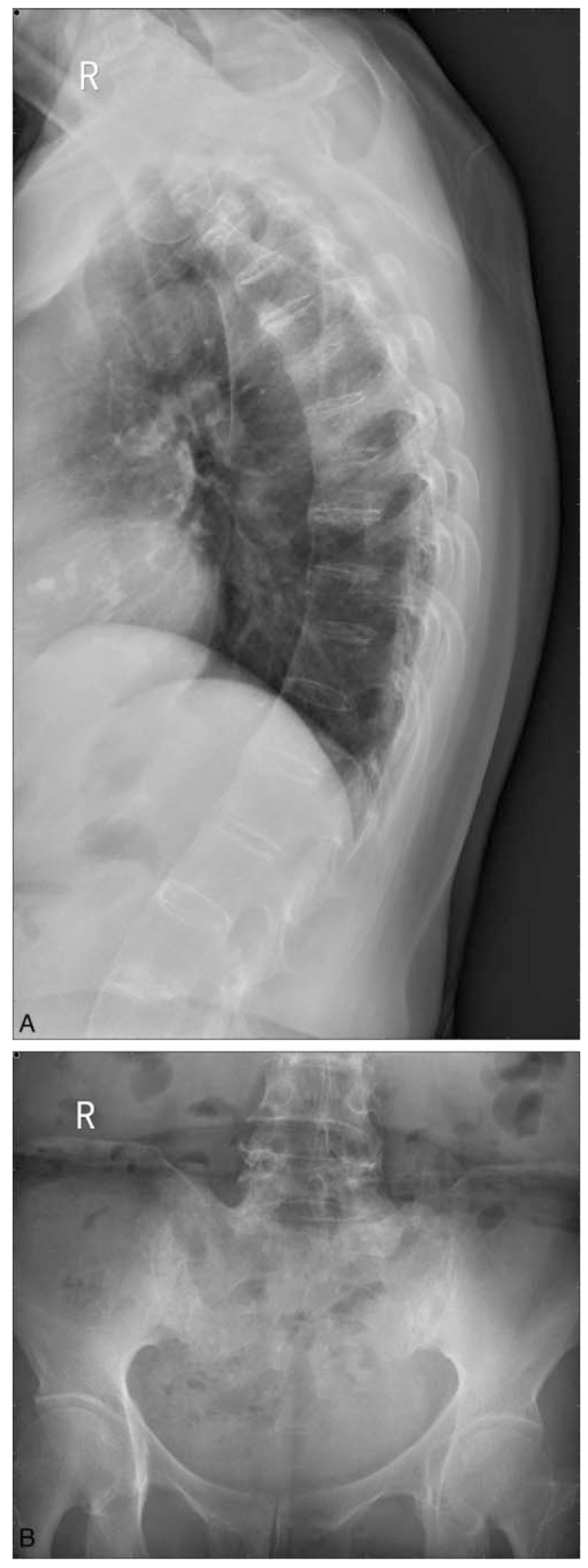
(A) Chest X-ray showed that the thoracic vertebrae underwent a bamboo-like change, but there was no narrowing of the intervertebral space. (B) Pelvic X-ray showed an increased density of bilateral sacroiliac joints and uneven joint gaps.

The patient was admitted with complaints of lumbosacral and bilateral knee pain, and morning stiffness lasting more than 90 minutes. The Bath Ankylosing Spondylitis Disease Activity Index (BASDAI) score was 6.08. Physical examination showed blood pressure of 152/97 mm Hg, pitting edema (grade 2) in bilateral lower extremities, and limited lumbar lateral flexion. Laboratory studies showed the following values: proteinuria (4+), urine protein quantitation (5.78 g/24 h), urinary microalbumin/creatinine (6,715 mg/g), serum creatinine (1.1 mg/dL), albumin (18.9 g/L), immunoglobulin IgA (3.75 g/L), and IgM (2.23 g/L), with globulin, IgG, complement C3 and C4, and serum-free k and λ within normal range. Serum phospholipase A2 receptor (PLA2R) antibodies were negative. Erythrocyte sedimentation rate was 48 mm/h. Antinuclear antibody was positive at a titer of 1:100, while double-stranded DNA and anti-glomerular basement membrane antibodies were negative. HLA-B27 was positive, chronic infectious diseases were negative, and cancer markers showed no clinically significant elevation. Chest X-rays suggested bamboo-like changes of the thoracic vertebrae. Pelvic X-ray suggested bilateral sacroiliitis (Grade III) (Fig. 1A, B).

**Figure 2:**
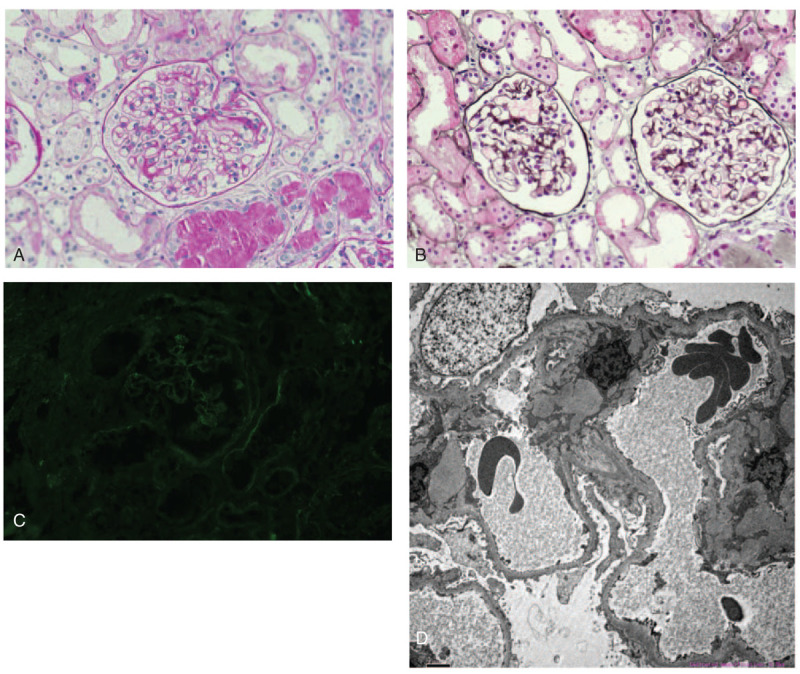
Renal biopsy. (A) Increased glomerular size and expanded capillary loop, PAS, 400 × ; (B) The thickening of the glomerular basement membrane, a small amount of red subepithelial fuchsinophilic deposits with spikes, PASM, 400 × ; (C) Renal phospholipase A2 receptor staining was negative, immunofluorescence 400 × ; (D) Electron microscopy showed swollen visceral epithelial cells with vacuolar degeneration, diffuse fusion of foot processes, and a small number of subepithelial electron-dense deposits.

The patient subsequently underwent a percutaneous renal biopsy. Light microscopy showed 16 glomeruli, with 2 of which showing glomerulosclerosis. The capillary loops of glomeruli were plump and thickened without proliferative activity or crescents. PASM and Masson staining demonstrated the thickening of the glomerular basement membrane and the presence of subepithelial fuchsinophilic deposits and spikes. Small focal (<25%) tubular atrophy, interstitial fibrosis, and mononuclear cell infiltration were present. Arteries showed mild intimal thickening (Fig. 2A, B). Using immunofluorescence staining, subepithelial deposits were visualized as fine granular positivity along the capillary wall. The deposits showed positive staining for IgG (++), slightly positive staining for IgG1 and IgM, and negative staining for IgG2, IgG3, IgG4, IgA, C3, C4, k, λ, and C1q. Renal PLA2R staining was negative (Fig. 2C). Evaluation using electronic microscopy showed subepithelial electron-dense deposits. The overlying podocyte foot processes were diffusely effaced. There were no evident changes in the mesangium or subendothelial area (Fig. 2D). All biopsy findings confirmed the diagnosis of MN and suggested secondary MN, likely a result of AS. The final diagnoses were AS, MN (probably secondary to AS), and thymoma (after resection).

During hospitalization, the patient's generalized edema became worse, Albumin decreased to 12 g/L, and creatinine rose to 1.5 mg/dL. Methylprednisolone (500 mg/d) was given for 3 consecutive days, followed by methylprednisolone 40 mg oral QD. One month later, the patient reported that her pain had disappeared completely. The microalbuminuria/creatinine decreased to 4606 mg/g, while albumin increased to 26 g/L.

The patient was followed up once a month. In the sixth month, the patient's serum creatinine decreased to 0.96 mg/dL, urine microalbumin/creatinine decreased to 173.3 mg/g, and albumin rose to 33.1 g/L. Pain and morning stiffness were relieved, and the BASDAI score dropped to 4.0.

## 3 Discussion

AS is a chronic autoimmune disease. Immune dysfunction of autoimmune diseases may lead to tumor development and progression.^[1]^ It has been reported that multiple autoimmune diseases, including myasthenia gravis, Crohn disease, ulcerative colitis, systemic lupus erythematosus, and silver psoriasis, are associated with cancers at multiple sites.^[7]^ Thymoma is closely associated with autoimmune diseases.^[8]^ The abnormally proliferating thymus cannot produce mature, properly functioning T cells. Deficiencies in T lymphocyte function may induce autoimmune diseases such as myasthenia gravis, erythropoiesis, systemic lupus erythematosus, inflammatory myopathy, and thyroid disease. Although there is no definite evidence linking AS and thymoma, 2 studies in Taiwan reported that the incidence of tumors in AS patients to be 1.15 times that of patients without AS.^[9]^ The incidence of gynecologic tumors, head and neck tumors, and bladder cancer in AS patients is 1.97 to 3.22 times that of non-AS patients. The risk of liver cancer and lung cancer is significantly higher in AS patients than in controls.^[10]^ Therefore, all patients with a definite diagnosis of AS should be screened for hematologic and solid tumors, including thymoma.

It was reported that renal involvement occurs in 21.7% (201/926) of AS patients.^[11]^ The most commonly reported renal complications are IgA nephropathy,^[12,13]^ followed by proliferative glomerulonephritis, secondary amyloidosis, and tubulointerstitial nephropathy.^[14,15]^ Long-term use of NSAID is a common cause of tubulointerstitial nephropathy.^[6]^ In AS patients, serum IgA is elevated,^[16]^ and IgA and immune complexes are deposited in the mesangial area, where they induce mesangial cells to produce pro-inflammatory cytokines such as IL-6, IL-1, MIP, and TNFα. These pro-inflammatory cytokines promote hyperplasia of the mesangial cells and extracellular matrix, which is an important mechanism of AS-induced glomerulonephritis, such as IgA nephropathy.^[14]^ MN in patients with AS has only rarely been reported.^[17–21]^ This patient was diagnosed with MN with routine pathological analysis, immunofluorescence microscopy, and also electron microscopy. First, idiopathic MN was ruled out because PLA2R antibodies, a specific marker of idiopathic MN,^[22]^ were absent from the serum and renal tissues. Second, except for the history of thymoma, which had been resected 1 year earlier, the screening results were negative for other causes of MN, including hepatitis B, systemic lupus erythematosus, and hematological and solid tumors. Although it has been reported that thymoma or thymic hyperplasia may lead to MN, renal injury is significantly improved after tumor resection,^[23]^ which did not happen in our reported case. For this reason, AS was considered to be more likely than thymoma to have given the patient's MN. As an autoimmune disease, AS is associated with a variety of autoantibodies, especially HLA B-27. The mechanism underlying MN in AS patients is still unclear. One hypothesis is that it has a mechanism similar to that of lupus nephritis, in which immune complexes of autoantibodies and autoantigens of glomerular epithelial cells activate the complement system and then trigger MN. However, this hypothesis requires further study.

Currently, there is no cure for AS. NSAIDS, DMARRDs, glucocorticoids, and biological agents are often used to treat symptoms and prevent disease progression. In this case, bilateral sacroiliitis was grade III and the BASDAI score was 6.08. After admission, the patient serum albumin decreased rapidly, and the renal function deteriorated rapidly. After glucocorticoid treatment, the renal functions (proteinuria and serum albumin) were improved and symptoms were relieved in 2 months.

In conclusion, this reported case had a 10-year history of AS. With intermittent NSAIDS treatment, the symptoms were only partially relieved, and the patient developed joint damage and renal complications. After treatment with glucocorticoids, joint pain, morning stiffness, and kidney function were significantly improved. Although the causal relationship between AS, thymoma, and MN in this patient still needs to be established, the genesis of the 3 diseases may have some association. In clinical practice, patients with AS should be screened for tumors and renal complications.

## Author contributions

**Data curation:** Lei Zhao, Luquan Zheng.

**Software:** Bengang Huo.

**Supervision:** Jurong Yang, Weibing Li.

**Validation:** Rongjie Yu.

**Writing – original draft:** Lirong Lin.

**Writing – review & editing:** Jurong Yang.
